# A Genomic Map of Climate Adaptation in *Arabidopsis thaliana* at a Micro-Geographic Scale

**DOI:** 10.3389/fpls.2018.00967

**Published:** 2018-07-10

**Authors:** Léa Frachon, Claudia Bartoli, Sébastien Carrère, Olivier Bouchez, Adeline Chaubet, Mathieu Gautier, Dominique Roby, Fabrice Roux

**Affiliations:** ^1^Laboratoire des Interactions Plantes-Microorganismes, Université de Toulouse, Institut National de la Recherche Agronomique, Centre National de la Recherche Scientifique, Castanet-Tolosan, France; ^2^Dipartimento di Biologia, Università degli Studi di Napoli Federico II, Naples, Italy; ^3^Department of Systematic and Evolutionary Botany, University of Zurich, Zürich, Switzerland; ^4^Institut National de la Recherche Agronomique, US 1426, GeT-PlaGe, Genotoul, Castanet-Tolosan, France; ^5^Centre de Biologie pour la Gestion des Populations, Institut National de la Recherche Agronomique, Centre de Coopération Internationale en Recherche Agronomique pour le Développement, Institut de Recherche pour le Développement, Montpellier SupAgro, Université de Montpellier, Montpellier, France

**Keywords:** *Arabidopsis thaliana*, Bayesian hierarchical model, climate change, genome–environment association analysis, local adaptation, Pool-Seq, spatial grain

## Abstract

Understanding the genetic bases underlying climate adaptation is a key element to predict the potential of species to face climate warming. Although substantial climate variation is observed at a micro-geographic scale, most genomic maps of climate adaptation have been established at broader geographical scales. Here, by using a Pool-Seq approach combined with a Bayesian hierarchical model that control for confounding by population structure, we performed a genome–environment association (GEA) analysis to investigate the genetic basis of adaptation to six climate variables in 168 natural populations of *Arabidopsis thaliana* distributed in south-west of France. Climate variation among the 168 populations represented up to 24% of climate variation among 521 European locations where *A. thaliana* inhabits. We identified neat and strong peaks of association, with most of the associated SNPs being significantly enriched in likely functional variants and/or in the extreme tail of genetic differentiation among populations. Furthermore, genes involved in transcriptional mechanisms appear predominant in plant functions associated with local climate adaptation. Globally, our results suggest that climate adaptation is an important driver of genomic variation in *A. thaliana* at a small spatial scale and mainly involves genome-wide changes in fundamental mechanisms of gene regulation. The identification of climate-adaptive genetic loci at a micro-geographic scale also highlights the importance to include within-species genetic diversity in ecological niche models for projecting potential species distributional shifts over short geographic distances.

## Introduction

In the context of contemporary climate change, a major goal in evolutionary ecology is to understand and predict the ability of a given species to persist in presence of novel climate conditions (Bay et al., 2017). A lack of response of species to selection due to climate change would cause an erosion of biodiversity by disrupting ecosystems sustainably (Pecl et al., 2017). Overall, species can adopt three non-exclusive responses to face the altered and fluctuating climate conditions (Aitken et al., 2008; Hansen et al., 2012; Pecl et al., 2017). Firstly, species can migrate to track current climate spatial shifts. This response can, however, be limited for (i) long-distance dispersal organisms because of the presence of multiple anthropogenic barriers (Ewers and Didham, 2006), and (ii) organisms with restricted dispersal as for example sessile plants lacking dispersal mechanism or disperser reward (Wang et al., 2016).

Secondly, organisms can rapidly acclimate to novel climate conditions *via* phenotypic plasticity, defined as the ability of a given genotype to produce different phenotypes when exposed to different environmental conditions (Fusco and Minelli, 2010). Despite its theoretical benefits to help natural populations to reach a new phenotypic optimum (Lande, 2009; Chevin et al., 2010), adaptive phenotypic plasticity is not as frequent as expected in nature because it can be constrained by several costs and limits (initially reviewed in DeWitt et al., 1998). For example, one of the main limits concerns the unreliability of environmental cues, leading to non-adaptive or mal-adaptive plastic responses (van Kleunen and Fischer, 2005). In the context of climate change, such unreliable cues can correspond to extreme climate events that fall outside the range of the climate conditions encountered by natural populations over their history (Orlowsky and Seneviratne, 2012).

Thirdly, over a longer term, organisms can adapt to novel climate conditions *via* genetic selection, provided that there is sufficient standing genetic variation or new genetic variation arising from either *de novo* mutations or immigration of climate-adapted alleles from nearby populations (Hoffmann and Sgrò, 2011; Bay et al., 2017). Predicting the response of species to climate change therefore requires the description of the genomic architecture (i.e., number of genes, allelic effects, locations across the genome) underlying climate adaptation. For this purpose, two major approaches can be adopted, based on either phenotype–genotype or ecology–genotype associations across the genome (Bergelson and Roux, 2010; Bay et al., 2017). Few genome-wide association mapping studies (GWAS) reported the genomic architecture associated with phenotypic traits potentially related to climate adaptation such as thermal sensitivity (Li et al., 2014). On the other hand, based on the assumption that each population is adapted to local environmental conditions, the most exploited approach corresponds to genome–environment association (GEA) analyses. In this case, a genome scan is performed to identify significant associations between genetic polymorphisms and environmental variables (Yoder et al., 2014; Abebe et al., 2015; Lasky et al., 2015; Rellstab et al., 2015; Hoban et al., 2016; Manel et al., 2016). Due to publicly available gridded estimates of climate and the development of next-generation sequencing (NGS) technologies, the number of GEA analyses performed on climate variables rapidly increased in the last few years (Bay et al., 2017). In most studies, the genomic architecture of climate adaptation was found to be highly polygenic, with hundreds to thousands of small-effect genetic variants scattered across the genome (Bay et al., 2017).

Most of the GEA analyses on climate were performed at large spatial scales (i.e., from several hundred to several thousand kilometers). However, substantial climate variation can also be observed at smaller spatial scales (from several tens of meters to several tens of kilometers), leading for example to sharp climate gradients in mountains (Manel et al., 2010; Kubota et al., 2015) or a mosaic of climatically optimal and suboptimal sites within the reach of gene flow among populations (Pluess et al., 2016). The complementarity of performing GEA analyses from continental to local geographical scales should shed light on the genetic bases underlying coarse-grained and fine-grained climate variation (Manel et al., 2010), which in turn would increase the reliability of predictions of response to climate change. In addition, as previously advised for adaptive phenotypic traits (Bergelson and Roux, 2010), working at a small geographical scale should reduce the limitations of GEA analyses often observed when working at larger geographical scales such as the confounding background produced by population structure, rare alleles and genetic/allelic heterogeneity. Finally, a fine-grained spatial scale is much more coherent with the mean distance of species migration (few km per decade; Chen et al., 2011).

*Arabidopsis thaliana* is a widely distributed annual selfing plant species found in a large range of climate environments across its native range in Eurasia (Hoffmann, 2002). Given the main barochorous mode of seed dispersal of *A. thaliana*, seeds are dispersed over short distances (i.e., a few ten of centimeters, Wender et al., 2005), thereby limiting the potential to track current climate poleward shifts. Although adaptive plastic responses to climate change (in particular seasonal climate change) have been observed in *A. thaliana* (Fournier-Level et al., 2016), a growing number of studies also reported the importance of genetic selection underlying climate adaptation in *A. thaliana* (Frachon et al., 2017). Notably, reciprocal transplants performed at the European scale revealed that climate gradients likely play a major role in local adaptation of *A. thaliana* (Fournier-Level et al., 2011; Ågren and Schemske, 2012; Ågren et al., 2013; Wilczek et al., 2014). Furthermore, amongst plant species, *A. thaliana* pioneered the identification of climate-adaptive genetic loci at the genome-wide scale. A GEA analysis based on 948 Eurasian accessions succeeded to establish a genomic map of local adaptation to climate variation, which in turn successfully predicted the relative fitness of a subset of accessions grown together in a common garden in the north of France (Hancock et al., 2011). Local adaptation to climate also explains a substantial portion of genomic variation of *A. thaliana* at a regional scale (i.e., several hundred km; Méndez-Vigo et al., 2011; Lasky et al., 2012; Vidigal et al., 2016; Tabas-Madrid et al., 2018). However, studies reporting the genomic architecture of adaptation to various climate variables at a finer spatial grain (from several tens of meters to several tens of kilometers) are still scarce in *A. thaliana*, despite the identification of strong climate–phenotype associations either along sharp altitudinal gradients (Montesinos-Navarro et al., 2011; Luo et al., 2015; Günther et al., 2016; Tabas-Madrid et al., 2018) or in a mosaic of climatically contrasted sites (Brachi et al., 2013).

In this study, we aimed to establish a genomic map of climate adaptation in *A. thaliana* at a micro-geographic scale. We focused on a new set of 168 natural populations distributed homogeneously in the south-west of France (Bartoli et al., 2018), a geographical region under the influence of three contrasted climates (i.e., oceanic climate, Mediterranean climate and mountain climate). By using a Bayesian hierarchical model that control for confounding by population structure (Gautier, 2015), we conducted a GEA analysis between 1,638,649 SNPs and six fine-grained climate variables. Because most *A. thaliana* natural populations located in France are genetically diverse (Le Corre, 2005; Platt et al., 2010; Brachi et al., 2013), we obtained a representative picture of within-population genetic variation across the genome by adopting a Pool-Seq approach. We then searched for genome-wide signatures of selection on the SNPs the most associated with climate variation. Following Hancock et al. (2011) and Brachi et al. (2015), we therefore tested whether those top SNPs were enriched either for non-synonymous variants or in the extreme tail of a genome-wide spatial differentiation scan. We finally discussed the function of the main candidate genes.

## Materials and Methods

### Plant Material

A field prospection in May 2014 allowed the identification of 233 *A. thaliana* natural populations in the Midi-Pyrénées region (south-west of France). In agreement with an important population turnover of natural populations observed in *A. thaliana* (Picó, 2012), individuals were present in only 168 populations (∼72.1%) in late winter 2015 when the sampling campaign was performed (Supplementary Table S1). The average distance among the 168 populations was 100.6 km (median = 93.4 km, max = 265.2 km, min = 0 km, first quartile = 57.3 km, third quartile = 137.2 km). To our knowledge, no natural populations of *A. thaliana* have been previously sampled in this geographic region.

### Climate Characterization

The 168 geo-localized populations were characterized for 20 biologically meaningful climate variables retrieved from the ClimateEU database (**Table 1**). Climate data has been generated with the ClimateEU v4.63 software package^1^ based on methodology described by Hamann et al. (2013). The grid resolution of the 20 climate variables (∼1.25 arcmin, ∼600 m) was smaller than the average distance among populations. Climate data were averaged across the 2003–2013 annual data. In addition, altitude was obtained from http://www.gps-coordinates.net. Following Hancock et al. (2011), the set of 21 climate variables was pruned based on the pairwise Spearman correlations of the variables (Supplementary Figure S1), by taking into account only variables that did not display a Spearman’s *rho* greater than 0.8. This step was performed to avoid inter-correlation between two variables. In cases where variables were strongly inter-correlated, we selected the variable with the most obvious link to the ecology of *A. thaliana*. The final set of six non-correlated climate variables considered in this study was composed by mean annual temperature, mean coldest month temperature and precipitations in winter, spring, summer, and autumn.

**Table 1:** List of the 21 climate variables used in this study.

Variable	Description	Source	Grid resolution^∗^
Altitude	Altitude (m)	www.coordonnees-gps.fr	–
**MAT**	**Mean annual temperature (°C)**	ClimateEU	1.25 arcmin
MWMT	Mean warmest month temperature (°C)	ClimateEU	1.25 arcmin
**MCMT**	**Mean coldest month temperature (°C)**	ClimateEU	1.25 arcmin
TD	Temperature difference between MWMT and MCMT, or continentality (°C)	ClimateEU	1.25 arcmin
MAP	Mean annual precipitation (mm)	ClimateEU	1.25 arcmin
AHM	Annual heat:moisture index (MAT + 10)/(MAP/1000)	ClimateEU	1.25 arcmin
SHM	Summer heat:moisture index [(MWMT)/(mean summer precipitation/1000)]	ClimateEU	1.25 arcmin
DD < 0	Degree-days below 0°C, chilling degree-days	ClimateEU	1.25 arcmin
DD > 5	Degree-days above 5°C, growing degree-days	ClimateEU	1.25 arcmin
DD < 18	Degree-days below 18°C, heating degree-days	ClimateEU	1.25 arcmin
DD > 18	Degree-days above 18°C, cooling degree-days	ClimateEU	1.25 arcmin
NFFD	The number of frost-free days	ClimateEU	1.25 arcmin
Tave_wt	Winter [December (previous year)–February] mean temperature (°C)	ClimateEU	1.25 arcmin
Tave_sp	Spring (March–May) mean temperature (°C)	ClimateEU	1.25 arcmin
Tave_sm	Summer (June–August) mean temperature (°C)	ClimateEU	1.25 arcmin
Tave_at	Autumn (September–November) mean temperature (°C)	ClimateEU	1.25 arcmin
**PPT_wt**	**Winter precipitation (mm)**	ClimateEU	1.25 arcmin
**PPT_sp**	**Spring precipitation (mm)**	ClimateEU	1.25 arcmin
**PPT_sm**	**Summer precipitation (mm)**	ClimateEU	1.25 arcmin
**PPT_at**	**Autumn precipitation (mm)**	ClimateEU	1.25 arcmin


*The final set of six non-correlated climate variables considered in this study are in bold. ^∗^1.25 arcmin ∼600 m.*

In order to compare the level of climate variation among the 168 populations of the Midi-Pyrénées region with the level of climate variation among natural populations of *A. thaliana* located in Europe, we first extracted data for the six climate variables (ClimateEU database) for 521 locations where *A. thaliana* have been collected and geo-localized in Europe, including 95 French locations (Hancock et al., 2011; Brachi et al., 2013). To visualize the climatic space encountered by *A. thaliana* at different geographical scales, we then performed a principal component analysis (PCA) based on the 689 locations by using the ade4 1.7-6 version package in the *R* environment (Chessel et al., 2004; Dray et al., 2017). Finally, the percentage of climatic variation in Europe observed among the 168 populations was calculated by dividing the extent of variation observed on the two first Principal Components (PC_climate_) at the scale of the Midi-Pyrénées region by the extent of variation observed at the European scale.

### Spatial Grains of Climatic Variation

To estimate the spatial grain of variation of each climate variable, a spectral decomposition of the spatial relationships among the 168 populations was first modeled with Principal Coordinates of Neighbor Matrices (PCNM), by running the pcnm() function implemented in the vegan package (R package version 2.3-5; Oksanen et al., 2016) using the Euclidean distance matrix based on the GPS coordinates of the 168 populations. This analysis allows decomposing the spatial structure among the sites under study into orthogonal PCNM components corresponding to successive spatial grains (Borcard and Legendre, 2002). The first PCNM components define a large spatial grain, while the last PCNM components correspond to finer grains (Borcard et al., 2004; Ramette and Tiedje, 2007). All PCNM components were then used as explanatory variables in a multiple linear regression on each climate variable. To account for multiple testing, a Benjamin–Hochberg procedure was performed for each climate variable across all PCNM components to control for a false discovery rate (FDR) at a nominal level of 5% (Benjamini and Hochberg, 1995).

### Genomic Characterization and Data Filtering

For each population, a mean number of 16.5 plants (total number = 2,776 plants, median = 17 plants, max = 17 plants, min = 5 plants, Supplementary Table S1) were collected randomly in late February – early March 2015 and brought back to a cold frame greenhouse (no additional light or heating). In April 2015, leaf tissue was collected from approximately 16 plants per population for a total of 2,574 plants (min = 5 plants, max = 16 plants, mean = 15.32 plants, median = 16 plants, Supplementary Table S1). More precisely, a portion of a rosette leaf for each plant was placed in 96-well Qiagen S-block plates containing a 3 mm bead in each well and samples were stored at -80°C. Prior to DNA extraction, plates were put 30 s in liquid nitrogen and samples were then crushed by using Mixer Mill MM 300 Retsch^®^ with 1 min of vibration at a frequency of 30 vibrations/s. Genomic DNA from the 2,574 plants was extracted as described in Brachi et al. (2013) and total DNA for each individual extraction was quantified by using a Quant-iT^TM^ PicoGreen^®^ dsDNA Assay Kit with a qPCR ABI7900 machine. Individuals from each population were then used to constitute an equimolar pool. From 50 to 500 bp fragments were produced for each pool by using Covaris M220 Focused-ultrasonicator^TM^ and fragment size selection was performed by using Sample Purification Beads. Produced fragments were analyzed with Agilent 2100 Bioanalyzer with a DNA 7500 chip and purified with Agencourt^®^ AMPure^®^ XP paramagnetic beads by following manufacturer instructions protocol. Illumina indexes were added by PCR amplification with the following cycling program: 1 min at 94°C, followed by 12 cycles of 1 min at 94°C, 1 min at 65°C and 1 min at 72°C, followed by a final elongation of 10 min at 72°C. After this step, PCR products were purified with Agencourt^®^ AMPure^®^ XP paramagnetic beads. The quality of the libraries was assessed with the Advanced Analytical Fragment Analyzer and libraries were then quantified by qPCR by using the Kapa Library quantification Kit. Samples were sequenced at the Get-PlaGe core facility (INRA of Toulouse) on an Illumina HiSeq 3000 sequencer by using a paired-end read length of 2 × 150 bp with the Illumina HiSeq3000 Reagents Kits. In particular, each Illumina lane was composed of a pool of 14 DNA samples. Twelve lines were used in total to sequence the DNAs (i.e., 14 *A. thaliana* populations were sequenced on each Illumina lane). Raw data for each population used in this study are available at the NCBI Sequence Read Archive (SRA)^2^ through the study accession SRP103198.

Raw reads were mapped on the reference genome Col-0 with glint tool (version 1.0.rc8.779) (Faraut and Courcelle, unpublished software) by using the following parameters: *glint mappe* –no-lc-filtering –best-score –mmis 5 –lmin 80 –step 2. The mapped reads were filtered for proper pairs with SAMtools (v0.01.19; Li et al., 2009) (*samtools view -f 0x02*). A semi-stringent SNPCalling across the genome was then performed for each population with SAMtools mpileup (Li et al., 2009) and VarScan mpileup2snp (Koboldt et al., 2012) software by using as parameters a minimum coverage (minimum read depth at a position to make a call) of five reads and a minimum variant allele frequency threshold of 0.00001. SNP-Pooling was then performed to obtain polymorphic sites across the pool of the 168 populations and SNP-Calling was inferred on the whole polymorphic sites as described above (VarScan mpileup2cns; min coverage = 1). The bi-allelic positions were then selected. The mean and the median coverage to a unique position in the reference genome was ∼26.3× and ∼24.5×, respectively (min = 11.60×, max = 48.69×).

After bioinformatics analysis, the allele read count matrix (for both the reference and alternate alleles) was composed by 4,781,661 SNPs across the 168 populations. This data set was further filtered using custom scripts. First, SNPs without mapped reads in at least eight populations were removed (number of remaining SNPs = 3,798,406). Second, for each population, we calculated the relative coverage of each SNP as the ratio of its coverage to the median coverage (computed over all the SNPs in the corresponding population). Because multiple gene copies in the 168 populations can map to a unique gene copy in the reference genome Col-0, we removed SNPs with a mean relative coverage across the 168 populations above 1.5 (number of remaining SNPs = 3,260,041). In addition, we removed SNPs with a standard deviation of allele frequency across the 168 populations below 0.004 (number of remaining SNPs = 3,248,168). Third, because genomic regions present in Col-0 can be absent in most of the 168 populations or genomic regions present in most of the 168 populations can be absent in Col-0, we removed SNPs with a mean relative coverage across the 168 populations below 0.5 (number of remaining SNPs = 3,172,313). Fourth, because of bias in GWA/GEA analysis due to rare alleles (Bergelson and Roux, 2010), we removed SNPs that were monomorphic in more than 90% of the populations, leading to a final data set consisting of read counts for 1,638,649 SNPs in the 168 populations.

### Genome–Environment Association Analysis

Based on the 1,638,649 SNPs, whole genome scans for adaptive differentiation and association with climate variables were performed with BayPass 2.1 (Gautier, 2015). Dealing with Pool-Seq data, the underlying Bayesian hierarchical models explicitly account for the scaled covariance matrix of population allele frequencies (Ω) which make the analyses robust to complex demographic histories.

Since the large number of SNPs analyzed here, we adopted a sub-sampling procedure to estimate Ω. This procedure consisted in dividing the full data set into 32 sub-data sets, each containing 3.125% of the 1,638,649 SNPs (51,207 SNPs taken every 32 SNPs along the genome), that were further analyzed in parallel under the core model using default options for the Markov Chain Monte Carlo (MCMC) algorithm (except -npilot 15 -pilotlength 500 -burnin 2500). Pairwise comparisons of the 32 resulting covariance matrices confirmed that all estimates were consistent with highly correlated elements. In addition, the pairwise FMD distances (Förstner and Moonen, 2003) had a narrow range of variation (from 2.04 to 2.24) with a mean value equal to 2.15.

These analyses carried out under the core model also provided the estimation of the XtX measure of differentiation for all the SNPs (combined over sub-data sets). For a given SNP, the XtX is defined as the variance of the standardized population allele frequencies, i.e., rescaled using Ω and across population allele frequencies (Günther and Coop, 2013; Gautier, 2015). This allows for a robust identification of highly differentiated SNPs by correcting for the genome-wide effects of confounding demographic evolutionary forces such as genetic drift and gene flow.

Given the close similarity of the Ω estimates obtained on the 32 sub-data sets, we retained for further analyses the matrix Ω^1 (obtained on the first sub-data set) as an estimate of the scaled covariance matrix of population allele frequencies. To evaluate the spatial scale of genomic variation, we first performed a singular value decomposition (SVD) of Ω^1. The coordinates of the two first resulting Principal Components (PC_genomic_) were then regressed against latitude and longitude of population *i* according to the following formula (PROC GLM procedure in SAS 9.3 SAS Institute Inc., Cary, NC, United States):

$${PC}_{genomic}{coordinates}_{i}-{latitude}_{i}+{longitude}_{i}+{latitude}_{i}^{*}{longitude}_{i}+\epsilon_{i}$$

Finally, genome-wide analysis of association with climate covariables were carried out under the AUX model (-auxmodel) parameterized with Ω_1_. The underlying model consists in a linear regression of the SNP population allele frequencies with the population-specific covariates while accounting for the shared covariance structure of allele frequencies as captured by the matrix Ω. In the AUX model, a Bayesian (binary) auxiliary variable δ is assigned to each SNP regression coefficient that indicates whether a specific SNP can be regarded as associated to the covariable (δ = 1) or not (δ = 0). By looking at the posterior mean of each SNP auxiliary variables (known as Posterior Inclusion Probability or PIP, in the model averaging literature), it is then straightforward to derive a Bayes Factor (BF) to compare models considering the SNP as associated or not (Gautier, 2015). Hence, the support for association of each SNP with each covariable *k* (i.e., a non-null regression coefficient β^ik between SNP *i* allele frequencies and a covariable *k*) was evaluated by computing BF measured in deciban units (dB) (Gautier, 2015). Note that the AUX model allows to explicitly accounting for multiple testing issue by integrating over (and estimating) the unknown proportion of SNPs actually associated with a given covariable. Here, six climate variables were considered separately and standardized prior to analyses using the scalecov option. In practice, BF and the associated regression coefficients β^i (mean of the product δ_i_β_i_ over the corresponding posterior sampled values) between SNP allele frequencies and climate variation were estimated for each SNP by analyzing in parallel the 32 sub-data sets described above (but with the same matrix Ω^1) using default options (except -npilot 15 -pilotlength 500 -burnin 2500) for MCMC.

### Testing the Power of GEA Analysis to Identify True Positives

Because climate variation was significantly associated with the first PC_genomic_ (Supplementary Table S2), we performed a simulation study aiming at characterizing how the degree of correlation between a given environmental covariable and the major axis of population structure might affect the sensitivity of BayPass to detect associated SNPs. For this purpose, using the R function *simulate.PCcorrelated.covariate* that we specifically developed in this study (Supporting Information of Supplementary Material) and that will be integrated in a future release of the BayPass software, we first simulated 10 environmental covariable vectors Zi (of length 168, the number of populations) with a Spearman’s coefficient correlation ρ_i_ ranging from 0 to 0.9 (ρ_i_ = 0.1(i-1) for i = 1,…, 10) with the first PC_genomic_. For each covariable vector, 25 data sets consisting of read counts for 10,000 SNPs including 100 associated SNPs (with a regression coefficient set at 0.1) were simulated under the BayPass inference model using the function simulate.baypass() available from the BayPass software package (Gautier, 2015). For all the simulated data sets, the simulation model was specified with the estimated matrix Ω^1 and the estimated posterior means of the two shape parameters of the Beta-distribution across population allele frequencies (∖hat{a} = 2.78 and ∖hat{b} = 0.710 with option pi.maf = 0.01). In addition, to preserve the characteristics of our design, we kept the same population haploid pool sizes and sampled the vector of SNP read coverages (across pool samples) from the observed data. The 250 resulting data sets were analyzed under the AUX model following standard procedure.

To compare the performances of the AUX model for different degrees of correlation of the underlying population-specific environmental covariable with the first PC_genomic_, the actual (i) true positive rates (TPRs) or power (i.e., the proportion of true positives among the truly associated SNPs); (ii) false positive rates (FPRs) (i.e., the proportion of false positives among the non-associated SNPs); and (iii) the false discovery rates (FDRs) (i.e., the proportion of false positives among the significantly associated SNPs) were computed for various thresholds covering the range of BF values (after combining, for each covariable vector, results on the 25 simulated data sets). From these estimates, both standard receiver operating curves (ROCs) plotting TPR against FPR and precision-recall (PR) curves plotting (1-FDR) against TPR were drawn. PR and ROC analyses were performed with the PRROC R package (Grau et al., 2015).

### Enrichment Across Annotation Categories of Variants for Climatic Associations

To test whether different categories of genetic variants were enriched for the SNPs that were the most significantly associated with climate variation (i.e., with the highest BF), we first annotated and predict the effect of all SNPs (*n* = 1,638,649 SNPs) by using the SnpEff program (Cingolani et al., 2012) in a Galaxy environment (Afgan et al., 2016). In this study, we considered six categories of genic variants representing 98.5% of the SNPs tested genome-wide, i.e., replacement variant (8.2%), synonymous variant (9.1%), intron variant (14.9%), UTR variant (5.6%), intergenic variant (49.1%), and intragenic variant (11.5%). For the intragenic variant category, 99.1% of the SNPs fall within transposable elements. We then tested whether SNPs in the 0.5% upper tail of the BF distribution of each climate variable were over-represented or under-represented in each of the six genetic variant categories. For each category of genetic variant, we used the following equation:

$${FE}_{SNPeff}=\frac{s_{a}/s}{S_{a}/S}$$

where *s* is the number of BF in the 0.5% upper tail of the BF distribution, *s*_a_ is the number of SNPs in the 0.5% upper tail of the BF distribution that also belonged to the genetic variant category, *S* is the total number of annotated SNPs tested genome-wide and *S*_a_ is the number of annotated SNPs tested genome-wide that also belonged to the genic variant category. Based on a methodology previously described in Hancock et al. (2011) that takes into account original Linkage Disequilibrium (LD) patterns among SNPs in the 0.5% upper tail of the BF distribution, statistical significance of enrichment for each category of genetic variants was assessed by running 10,000 null circular permutations of SNPs along the genome.

### Enrichment in Signatures of Selection for Climatic Associations

For each climate variable, we tested whether SNPs with the highest BF were over-represented in the extreme tail of the XtX distribution according to the methodology described in Brachi et al. (2015):

$${FE}_{XtX}=\frac{n_{a}/n}{N_{a}/N}$$

where *n* is the number of XtX in the 0.5% upper tail of the XtX distribution, *n_a_* is the number of SNPs in the 0.5% upper tail of the BF distribution that were also in the 0.5% upper tail of the XtX distribution, *N* is the total number of SNPs tested genome-wide and *N_a_* is the number of SNPs in the 0.5% upper tail of the BF distribution. Statistical significance of enrichment was assessed by running 10,000 null permutations based on the methodology described in Hancock et al. (2011).

### Identification of Candidate Genes Associated With Climate Variation

A three-step procedure was adopted to identify candidate genes associated with climate variation. Firstly, we selected the 50 SNPs with the highest BF for each of the six climate variables, leading to a total of 300 SNPs. Secondly, for each climate variable, candidate regions were defined on the genomic regions supported by at least three top SNPs successively separated by less than 10 kb. This step led to the identification of 12 candidate regions, including one region detected for both mean annual temperature and mean coldest month temperature. The 12 candidate regions contain on average 3.6 SNPs (median = 4 SNPs, min = 3 SNPs, max = 6 SNPs) and have a mean length of 10.1 kb (median = 9.5 kb, min = 2.4 kb, max = 16.9 kb). Finally, using the TAIR 10 database^3^ we retrieved all the annotated genes located within or overlapping with the 13 candidate regions, leading to the identification of 34 annotated genes.

## Results

### Climate Variation and Associated Spatial Grains

**FIGURE 1:**
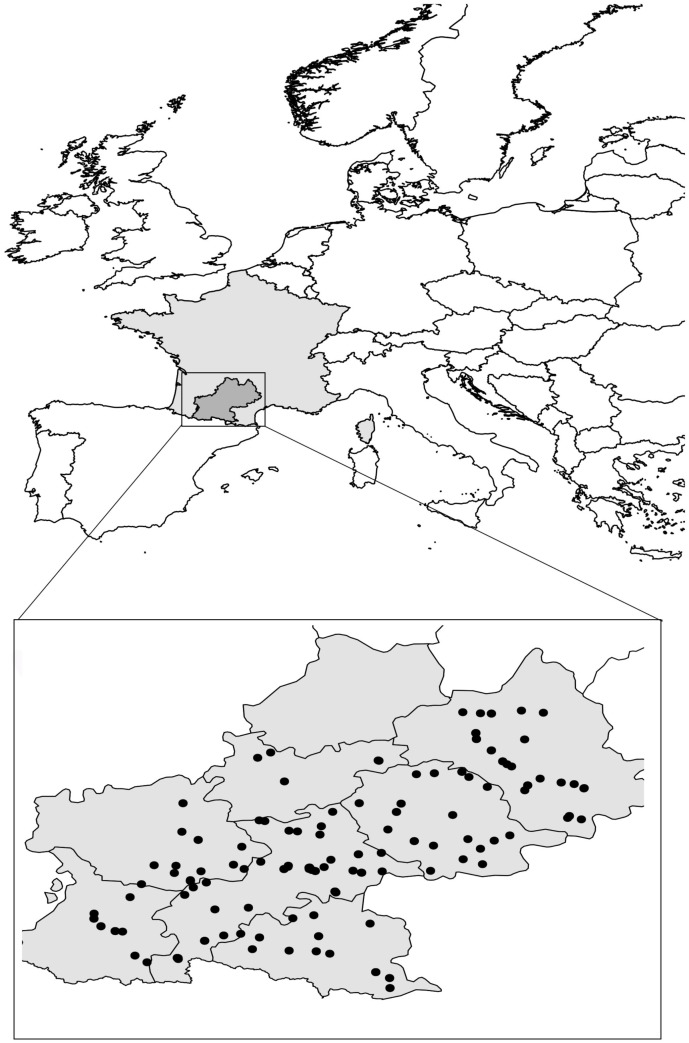
Distribution of the 168 *A. thaliana* natural populations across the Midi-Pyrénées region (south-west of France). Gray zone represents total area of metropolitan France. Black dots represent locations inhabited by *A. thaliana* in the Midi-Pyrénées region.

Here, we focused on 168 *A. thaliana* natural populations distributed in the Midi-Pyrénées region located in the south-west of France (**Figure 1**). We identified 82 Principal Coordinates of Neighbor Matrices (PCNM) components, suggesting a relatively homogeneous spatial distribution of the 168 populations across the sampling area (**Figure 1**).

The 168 populations were characterized for six climate variables with a grid resolution (∼600 m) smaller than the average distance among populations (i.e., 100.6 km, *SD* = 56.0 km). The six climate variables were associated with a large range of PCNM components, (Supplementary Figure S2), indicating coarse-grained to very fine-grained spatial variation for temperature- and precipitation- related variables.

**FIGURE 2:**
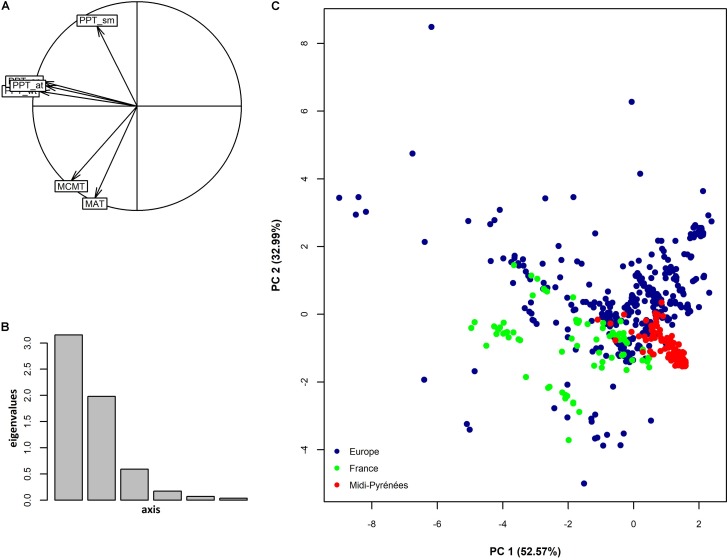
Climate variation among natural populations of *A. thaliana* collected at different geographical scales. **(A)** Factor loading plot resulting from a principal component analysis. Factor 1 and factor 2 explained 52.57% and 32.99% of total climate variation. See **Table 1** for a description of the climate variables. **(B)** Distribution of eigenvalues. **(C)** Position of the 168 populations of the Midi-Pyrénées region in the European and French climatic space of *A. thaliana*. Blue dots represent European locations without considering locations in France (*n* = 426), green dots represent French locations without considering locations in the Midi-Pyrénées region (*n* = 95), red dots represent locations in the Midi-Pyrénées region (*n* = 168).

Despite the restricted size of our sampling area (∼8.2% of total area of metropolitan France, **Figure 1**), climate variation among the 168 populations represented 24.0% and 14% of climate variation observed among 521 European locations for the first and second PC_climate_, respectively (**Figure 2**). In addition, the climate space of the Midi-Pyrénées region largely differed from the climate space of the other French regions inhabited by *A. thaliana* (**Figure 2C**).

Altogether, these results suggest the presence of contrasted climates, even at a short geographical distance, among the locations inhabited by *A. thaliana* in the Midi-Pyrénées region.

### Genome–Climate Associations

Using Pool-Seq data, we estimated within-population allele frequencies across the genome for a final number of 1,638,649 SNPs (i.e., one SNP every 72 bp). Based on SVD of the population covariance-variance matrix Ω^1, we found that 96.4% of the genomic variation observed in the Midi-Pyrénées region was explained by the first PC_genomic_ (Supplementary Figure S3), reinforcing the pattern of strong population subdivision already observed in other French regions (Le Corre, 2005; Brachi et al., 2013). In addition, a weak geographic pattern along a south-west/north-east axis was observed for genomic variation (latitude: *t* value = 4.734, *P* = 4.73 × 10^-6^, longitude: *t* value = 4.417, *P* = 1.81 × 10^-5^, latitude × longitude: *t* value = -4.428, *P* = 1.73 × 10^-5^, adjusted *R*^2^ = 10.5%; Supplementary Figure S3).

**FIGURE 3:**
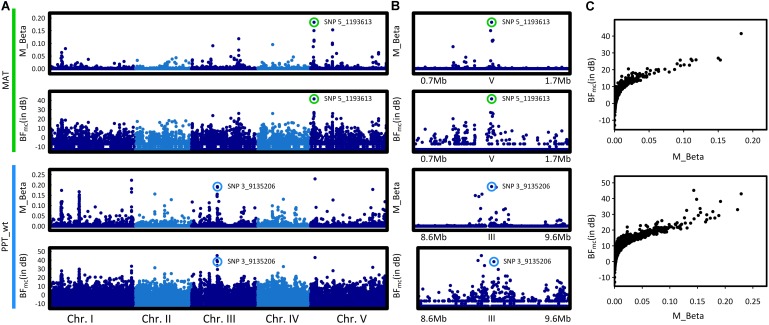
Manhattan plots of the genome–environment association results for mean annual temperature (MAT) and winter precipitations (PPT_wt). **(A)** The *x*-axis indicates the position along each chromosome of the 1,638,649 SNPs. The five chromosomes are presented in a row along the *x*-axis in different shades of blue. The *y*-axis indicates either the posterior mean of the absolute regression coefficient β_i_ (M_Beta value) or the Bayes factor (BF_mc_ expressed in deciban units), estimated by the AUX model. Colored circles highlight the SNP with the highest absolute regression coefficient β_i_ within the most significant association peak. **(B)** Zooms spanning the genomic regions in which the SNP with the highest BF_mc_ value is located. Colored circles highlight the SNP with the highest absolute regression coefficient β_i_ within the most significant association peak. **(C)** Estimates of the Bayes factor as a function of the absolute regression coefficients (β_i_).

**FIGURE 4:**
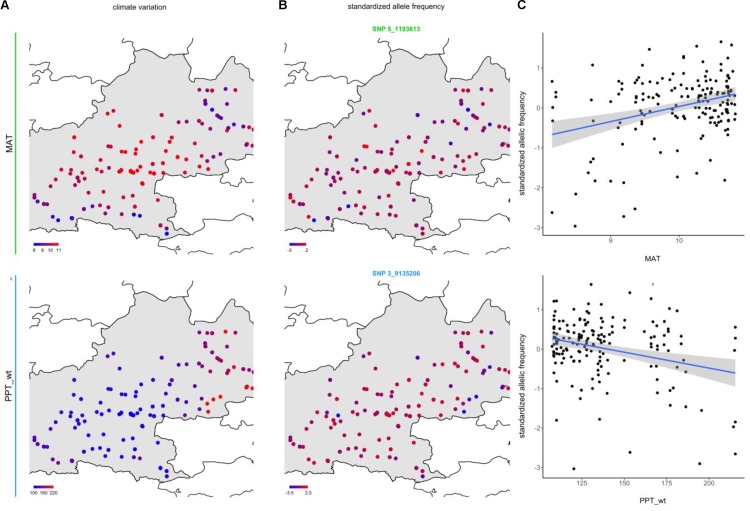
Relationships between climate variation and allele frequency variation at candidate SNPs for mean annual temperature (MAT) and winter precipitations (PPT_wt). **(A)** Map illustrating the geographic variation of MAT and PPT_wt. **(B)** Map illustrating the geographic variation of the standardized allele frequencies of one of the SNPs the most associated with these climate variables (see **Figure 3B**). **(C)** Bi-plots illustrating the relationships between standardized allele frequency variation (SNP_5_1193613 for MAT and SNP 3_9135206 for PPT_wt) and climate variation. The blue line and its associated gray area correspond to the standardized allele frequency variation – climate variation linear fit and its associated 95% confidence intervals, respectively.

To identify the genomic regions associated with climate variation, we then performed a genome-wide scan for association with the six climate variables, using a Bayesian hierarchical model that includes a population covariance matrix accounting for the neutral covariance structure across population allele frequencies. For each climate variable, we estimated the regression coefficients between SNP allele frequencies and climate variation (β_i_) and evaluated the support for association (non-null β_i_) of the association between a given SNP and a climate variable with a Bayes factor (BF). By applying this method, we identified neat and strong peaks of association for most of the climate variables (**Figure 3** and Supplementary Figure S4). Accordingly, as illustrated for the mean annual temperature and the winter precipitations, standardized allele frequencies variation of the most significant SNPs strongly overlapped with climate variation (**Figure 4**).

**FIGURE 5:**
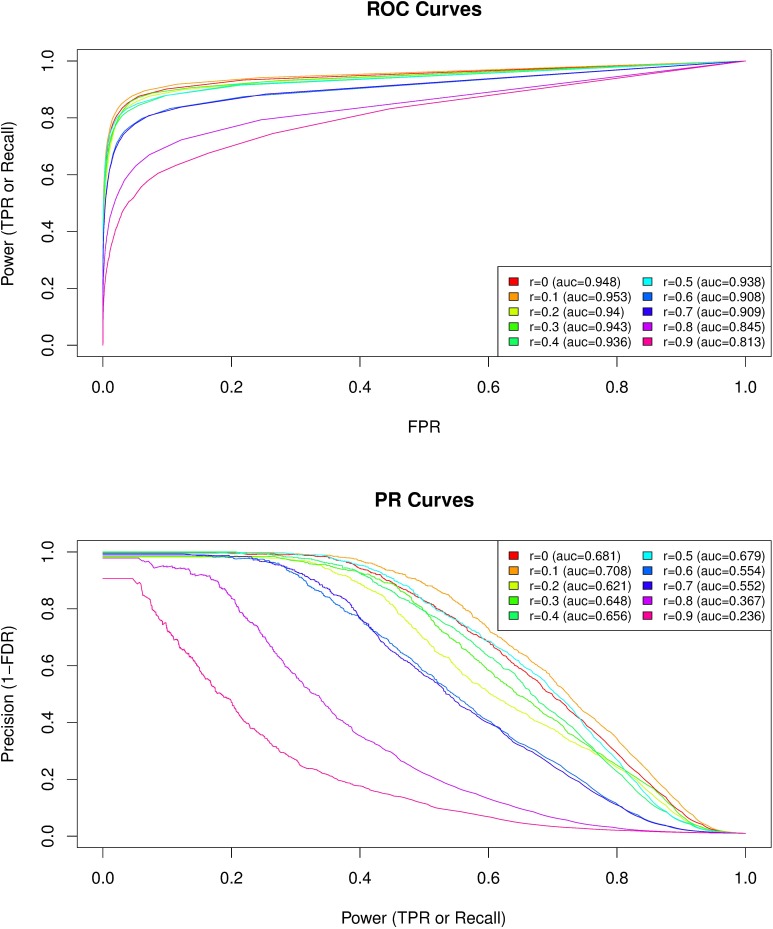
Comparison of the performances of BayPass for the identification of SNPs associated with population-specific covariable with varying correlation coefficients with the first axis of genetic variation PC_genomic_. For each of the 10 covariable considered with correlation coefficients ranging from Spearman’s *rho* = 0 to Spearman’s *rho* = 0.9 (with an increasing step of 0.1) with the first PC_genomic_, the plotted average ROC **(Upper)** and PR **(Lower)** curves result from the analyses of the 25 simulated data sets consisting of 10,000 SNPs including 100 associated SNPs (β = 0.1). The simulation model was calibrated to obtain data sets closely mimicking our observed data both in terms of the sample design (number of populations, haploid sample size of the pools, read coverage, across population allele frequencies) and population structure (summarized by the matrix Ω). The corresponding area under the curve (AUC) are reported in the box legend of each plot (r, Spearman’s *rho*).

Because climate variation was significantly associated with the first PC_genomic_ (absolute Spearman’s correlation coefficients ranging from 0.148 to 0.316; Supplementary Table S2), we evaluated to which extent the level of correlation of the analyzed covariables with this axis can have affected the performance of GEA analyses. We analyzed 250 data sets simulated under the inference model and each data set consisted of 10,000 SNPs including 100 SNPs associated with a given environmental covariable out of 10 simulated ones (25 data sets per covariable) that displayed different levels of correlation with the first PC_genomic_ (ranging from 0 to 0.9 with an increasing step of 0.1). The simulation model was calibrated to obtain data sets closely mimicking our observed climate data, both in terms of the sample design (number of populations, haploid sample size of the pools, read coverage, across population allele frequencies) and population structure (summarized by the matrix Ω). As expected, performances of the model for detecting environmental covariable – SNPs associations decreased (i.e., for a given power both the false discovery and false positive rates increased) when the correlation of the considered environmental covariable with the first PC_genomic_ increased (**Figure 5**). For instance, ROC and PR AUC were minimal for Spearman’s *rho* = 0.9 (ROCauc = 0.813 and PRauc = 0.236) (**Figure 5**). Interestingly, up to correlation values equal to 0.5 (i.e., beyond the range of our climate variables of interest), performances of the model remained similar, with ROC AUC ranging from 0.936 (Spearman’s *rho* = 0.4) to 0.953 (Spearman’s *rho* = 0.1) and PR AUC ranging from 0.629 (Spearman’s *rho* = 0.2) to 0.701 (Spearman’s *rho* = 0.1) (**Figure 5**). Altogether, these results suggest that a large fraction of the SNPs the most associated with climate variation correspond to true positives.

### Signatures of Selection

**FIGURE 6:**
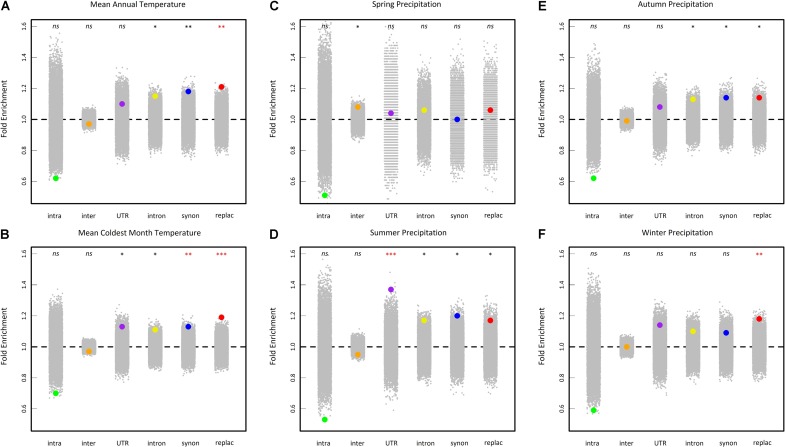
Enrichment analysis of different classes of sites in the 0.5% upper tail of the BF_mc_ distribution of six climate variables (**A**: mean annual temperature, **B**: mean coldest month temperature, **C**: spring precipitation, **D**: summer precipitation, **E**: autumn precipitation, and **F**: winter precipitation). Enrichments shown are relative to the proportion of each class of SNPs in the genome overall. Large dots represent the enrichment values for each class of sites. Gray dots represent 10,000 null permutations of site categories. The horizontal dashed line shows the expected enrichment under the null hypothesis of no enrichment. Enrichments that are significant relative to permutations are denoted by asterisks. ^ns^non-significant, ^∗^0.05 > *P* > 0.01, ^∗∗^0.01 > *P* > 0.001, ^∗∗∗^*P* < 0.001. Red asterisks indicate significant enrichments after a false discovery rate (FRD) correction at the nominal level of 5%. intra, intragenic SNPs (i.e., SNPs in transposable element gene); inter, intergenic SNPs; UTR, SNPs in 5′ and 3′ untranslated transcribed regions; intron, intronic SNPs; synon, synonymous SNPs; replac, replacement SNPs (amino-acid changing SNPs and stop codon gained SNPs).

To test whether our results detect true signals of adaptation, we first tested whether the top SNPs (i.e., the 0.5% upper tail of the BF distribution) were differentially enriched for six categories of genetic variants (intragenic, intergenic, UTR, intron, synonymous and replacement). For the variables mean annual temperature, mean coldest month temperature and winter precipitations, the highest significant fold enrichment was observed for replacement SNPs (**Figure 6**). For the variable summer precipitations, the highest significant fold enrichment was detected for SNPs located in UTR regions (**Figure 6**). No significant enrichment was detected for spring and autumn precipitations variables after a correction for multiple comparisons (**Figure 6**).

We then tested whether the top SNPs (i.e., the 0.5% upper tail of the BF distribution) significantly overlapped with the 0.5% upper tail of the XtX distribution (i.e., the 0.5% most overly differentiated SNPs among populations). For all climate variables with the exception of winter precipitations, climate-related SNPs were significantly enriched in the 0.5% upper tail of the XtX distribution (*P* < 0.001), with a fold-enrichment ranging from 1.70 for mean coldest month temperature to 3.14 for spring precipitations.

### Identification of Candidate Genes

To identify candidate genes associated with climate variation, we retrieved all the annotated genes located within or overlapping with 12 candidate regions (each supported by at least three top SNPs), including one region at the beginning of chromosome 5 detected for both mean annual temperature and mean coldest month temperature (Supplementary Table S3). By considering a list of 34 candidate genes, a literature survey identified different functions encoded by these genes that can be classified in two main categories : (i) a large number of proteins (*n* = 16) involved in the regulation of gene expression, including RNA binding proteins and transcriptional factors, but also miRNAs or transposable elements; (ii) genes involved in abiotic (or biotic) stress response (*n* = 6), including genes involved in the regulation of salt stress, cold/high temperature stress, light/dark treatment, or pathogen attack such as ACR8, RZ-1C, AN1 like zing finger protein, MAF1/FLM or extensin-like protein (ELP). Other candidate genes correspond to diverse general functions, including developmental regulators, or to unknown functions.

## Discussion

### Climate Adaptation in a Patchy Climate Environment

In agreement with the influence of three contrasted climates (i.e., oceanic climate, mountain climate, and Mediterranean climate) in the south-west of France, up to 24% of climate variation across European locations inhabited by *A. thaliana* was observed in the Midi-Pyrénées region. The presence of mountains in the south and north-east in our sampling area likely explained the steep temperature gradients observed in this study. On the other hand, we observed a mosaic of contrasted precipitation regimes. Therefore, the different spatial grains between temperature and precipitations lead to rugged climate landscapes over very short geographical scales, which in turn better match with the small distance of seed and pollen dispersal expected for a barochorous and selfing plant species such as *A. thaliana*.

In comparison with studies performed at larger geographical scales (Hancock et al., 2011; Lasky et al., 2012), we identified very neat and strong peaks of association with climate variation. Such a pattern is similar to a previous GWAS in *A. thaliana* reporting that the significance level of association peaks for phenological traits potentially related to climate adaptation (such as flowering time) was stronger based on regional or local accessions than worldwide or European accessions (Brachi et al., 2013). Two non-exclusive hypotheses can be suggested to explain the strong SNP-climate associations detected in our study. First, because the genetic variability of most natural populations of *A. thaliana* has long been considered to be low (likely due to its selfing rate close to 98%; Platt et al., 2010), most genome–climate association studies performed at the European or regional scale have been based on very few accessions per population, i.e., ∼1.7 accession per population (Hancock et al., 2011; Lasky et al., 2012). However, recent studies challenged this view, and many natural populations have been described to be highly genetically variable at both neutral SNPs and polymorphisms associated within natural phenotypic variation (Le Corre, 2005; Picó et al., 2008; Lundemo et al., 2009; Montesinos et al., 2009; Bomblies et al., 2010; Platt et al., 2010; Kronholm et al., 2012; Samis et al., 2012; Brachi et al., 2013; Huard-Chauveau et al., 2013; Karasov et al., 2014; Luo et al., 2015; Debieu et al., 2016; Frachon et al., 2017). Since pool sequencing has been demonstrated a cost-effective method to infer demography and to identify genetic markers underlying local adaptation in several plant and animal species (Schlötterer et al., 2014), we obtained a representative picture of within-population genetic variation across the genome by sequencing pools of ∼16 individuals from each population. Second, in agreement with previous studies reporting that global effects of the demographic evolutionary forces in *A. thaliana* should be limited at a small geographical scale (Nordborg et al., 2005; Platt et al., 2010), we observed that genomic variation among the 168 natural populations is weakly correlated to geographic variation. Such a pattern likely alleviated the limitations of GEA analyses often observed at larger geographical scales such as confounding background produced by population structure, rare alleles and genetic/allelic heterogeneity.

Importantly, following methodologies previously developed in *A. thaliana* to identify environment-adaptive genetic loci at the European scale (i.e., climate and herbivore resistance; Hancock et al., 2011; Lasky et al., 2012, 2018; Brachi et al., 2015), we found that the SNPs the most associated with climate were significantly enriched in likely functional variants (i.e., non-synonymous variants) and/or in the extreme tail of spatial differentiation among populations. These clear signatures of selection suggest that climate is an important driver of adaptive genomic variation in *A. thaliana* at a micro-geographic scale. Although studies reporting the identification of climate adaptive genetic loci at a small geographical scales are still scarce (Manel et al., 2010; Kubota et al., 2015; Günther et al., 2016; Pluess et al., 2016), there is mounting genomic evidence that micro-geographic adaptation to climate is more widespread than is commonly assumed.

### Overrepresentation of Genes Involved in Regulatory Mechanisms in the Plant Functions Involved in Local Adaptation to Climate

The identification of candidate genes associated with climate variation suggests that regulation of gene expression is a key factor of climate adaptation. Transcription factors (TFs), RNA binding proteins and epigenetic mechanisms were the major functions uncovered by our study. These findings are in agreement with global gene expression studies demonstrating that plant response to environmental constraints relies on a number of distinct transcriptional responses operating spatially, temporally and in combination with other signals like hormones (Coolen et al., 2016). The *RZ-1C* protein identified here in association with the mean annual temperature and the mean coldest month temperature, was shown to belong to a unique group of GRPs (Glycine-rich RNA binding Proteins) with potential roles in increasing cold tolerance in Arabidopsis (Kim et al., 2010). These proteins have been recently shown to control gene splicing *via* interaction with SR (Serine/arginine-Rich) proteins, and consequently expression of many genes, including developmental regulators. Among them, the MADS-box transcription factor *FLOWERING LOCUS C* (*FLC*) is a direct target of *RZ-1C* that both promotes its splicing and represses its transcription (Wu et al., 2016). In the same vein, the MADS box transcription factor gene *FLOWERING LOCUS M* (*FLM/MAF1*) is involved in temperature dependent regulation of flowering through *FLM* splicing changes and nonsense-mediated mRNA decay in response to elevated temperature (Posé et al., 2013; Sureshkumar et al., 2016). miR172b, also found here in relation with the mean annual temperature and the mean coldest month temperature, is a particularly interesting candidate as it controls transition of germinating seedlings from heterotrophic to autotrophic growth, and consequently the post-germination developmental arrest checkpoint under diverse stress (Zou et al., 2013). Found in relation to spring precipitations, *ACT domain repeat 8* (*ACR8*) belongs to the *ACR* gene family which is differentially regulated by diverse abiotic stress (salt, cold, light/dark) and plant hormones. Interestingly, ACR proteins bind to specific ligands (amino acids or small ligands) and are thought to exert their regulatory functions through this binding and/or by sensing environmental conditions (Hsieh and Goodman, 2002). These examples illustrate the importance of diverse regulatory processes (metabolic and hormonal signals, RNA splicing, gene expression, protein-protein interactions, …) in adaptation to climate variables. Interestingly, we also identified some transposable elements (known to be related with gene regulation) as candidate genes. This is in agreement with a recent work demonstrating that the composition and the activity of the Arabidopsis mobilome vary greatly among accessions (Quadrana et al., 2016) and that loci controlling adaptive responses to the environment are the most frequent transposition targets observed.

## Conclusion

In agreement with the increasing number of phenotypic studies reporting micro-geographic adaptation (Richardson et al., 2014), climate variation appears as an important driver of adaptive genomic variation in *A. thaliana* at a fine spatial grain. This result reinforces the need to choose mapping populations according to the spatial scale of ecological variation at which species are adapted (Bergelson and Roux, 2010). In addition, the identification of climate-adaptive genetic loci at a micro-geographic scale highlights the importance to include within-species genetic diversity in ecological niche models for projecting potential species distributional shifts over short geographic distances (Valladares et al., 2014). The over-representation of genes involved in regulatory mechanisms in the plant functions associated with climate variation is a common pattern at different geographical scales in *A. thaliana*. The candidate genes identified in this study undoubtedly constitute key candidate genes for functional analysis, thereby providing an exciting opportunity to dissect the molecular bases of climate adaptation at a short geographic scale. The most significant associated SNP identified here for the mean cold month temperature in the *RZ-1C* gene is of particular interest because it leads to an amino acid change (Tyr → Phe) in a highly conserved domain of 25 amino acids in the Brassicaceae family (Supplementary Figure S5).

## Author Contributions

LF, CB, and FR planned and designed the research. LF and FR conducted fieldwork. LF performed the climate database searches. CB and FR performed the DNA extraction. CB, OB, and AC generated the sequencing data. CB and SC performed the bioinformatics analysis. LF and MG performed the genome–environment association analysis. LF, DR, and FR performed the enrichment analyses and the identification of candidate genes. LF, CB, MG, DR, and FR wrote the manuscript. All authors contributed to the revisions.

## Conflict of Interest Statement

The authors declare that the research was conducted in the absence of any commercial or financial relationships that could be construed as a potential conflict of interest.

## Footnotes

1 http://tinyurl.com/ClimateEU

2 http://ncbi.nlm.nih.gov/sra

3 https://www.arabidopsis.org/
