# Supplementary material for: A Genomic Map of Climate Adaptation in Arabidopsis thaliana at a Micro-Geographic Scale
Source: Front Plant Sci. 2018 Jul 10;9:967. doi: 10.3389/fpls.2018.00967 (PMC6048436; doi:10.3389/fpls.2018.00967)
Supplement: Supplementary file 2 [file Data_Sheet_1.DOCX]

**Supporting Information**

**A genomic map of climate adaptation**

**in *Arabidopsis thaliana* at a micro-geographic scale**

**Léa Frachon, Claudia Bartoli, Sébastien Carrère, Olivier Bouchez, Adeline Chaubet, Mathieu Gautier, Dominique Roby and Fabrice Roux^*^**

*** Correspondence:** Corresponding Author: fabrice.roux@inra.fr

3 Supplementary Tables

5 Supplementary Figures

**Supporting Information: Function to simulate covariate value correlated to a given Omega PC**

simulate.PCcorrelated.covariate <- function(omega,axis=1,targeted.rho=0.1,tol=0.01){

npops=nrow(omega)

om.svd=svd(omega)

PC=om.svd$u[,axis]

PC.scaled=scale(PC)

###hybrid ALGO between brut force (since PC not necessarily gaussian) and projection

#See https://stats.stackexchange.com/questions/15011/generate-a-random-variable-with-a-defined-correlation-to-an-existing-variable

tol=tol*abs(targeted.rho)

target.min=max(-1,targeted.rho-tol)

target.max=min(1,targeted.rho+tol)

C = matrix(targeted.rho,2,2)

diag(C) = 1

C=chol(C)

cc=100 ; cnt=0

while(cc<target.min | cc>target.max){

nn=(cbind(PC.scaled,rnorm(npops))%*% C)[,2]

cc=cor(PC,nn)

cnt=cnt+1

}

cat(targeted.rho," found in ",cnt," iterations\n")

return(nn)

}

**Table S1. Names and GPS coordinates (expressed in decimal degrees) of the 168 populations.**

**^1^ number of plants collected randomly in each population, ^2^ number of plants used for DNA extraction in the Pool-Seq approach.**

**Table S1 (continued)**

**Table S1 (continued)**

**Table S1 (continued)**

**Table S2 Relationships between climate variation and the first Principal Component obtained after a singular value decomposition of the scaled covariance matrix of population allele frequencies** $\hat{\boldsymbol{\Omega}_{\boldsymbol{1}}}$. *rho* : Spearman’s *rho*. MAT: mean annual temperature, MCMT: mean coldest month temperature, PPT_sp: spring precipitations, PPT_sm: summer precipitations, PPT_at: autumn precipitations, PPT_wt; winter precipitations.

**Table S3 List of the annotated genes located within or overlapping with 12 candidate regions.** ‘QTL number’ correspond to the 12 candidate regions, each being supported by at least three top SNPs successively separated by less than 10kb (NB: a candidate region was found both for MAT and MCMT). ‘climate factor’ corresponds to the six climate variables (see Table 1 for a description of the climate variables). ‘chromosome’ and ‘position’ stands for the physical positions of the 50 top SNPs for each of the six climate variables. ‘BF(db)’: Bayes factor (BF_mc_ expressed in deciban units) estimated by the AUX model. Values in red highlight the SNP with the highest BF_mc_ value within each candidate region. ‘Atg number’: Atg numbers in red correspond to genes in which the SNP with the highest BF_mc_ value in a given candidate region is located. When the SNP with the highest BF_mc_ value in a given candidate region is located in an intergenic region, the two adjacent genes are highlighted in red.

**Figure S1. Correlation matrix among the 21 climate variables.** Above diagonal: values of Spearman’s *rho*. Below diagonal: levels of significance values of Spearman’s *rho* after a false discovery rate (FDR) correction at the nominal level of 5%. See Table 1 for a description of the climate variables.

**Figure S2.** **Spatial grains of climate variation based on multiple regressions of each of the six climate variables on 82** **Principal Coordinates of Neighbour Matrices (PCNM) components**. The significant regression coefficients are colored by a blue gradient after a false discovery rate (FDR) correction at the nominal level of 5% (light blue * *P* < 0.05, medium blue ** *P* < 0.01, dark blue *** *P* < 0.001). The PCNM components for which no significant regression coefficient was detected are not represented. See Table 1 for a description of the climate variables.

**
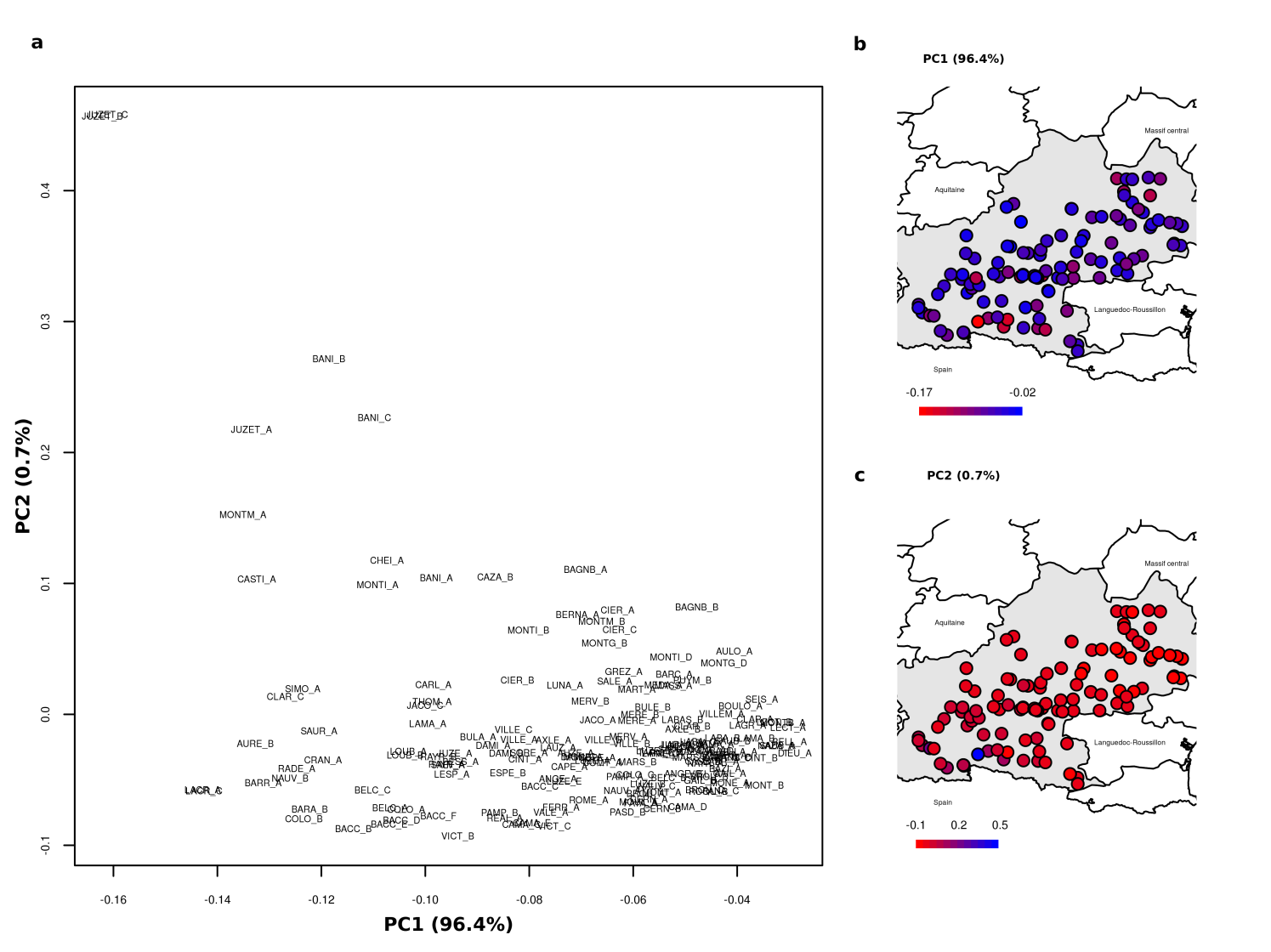
Figure S3. Spatial scale of genomic variation among the 168 populations of *A. thaliana*.** (**a**) Singular value decomposition (SVD) run on the covariance-variance matrix obtained with the first sub-data set of 51,208 SNPs. (**b**) and (**c**) Geographic map of the coordinates of the 168 populations on the first and second Principal Components, respectively.

**Figure S4. Relationship between the posterior mean of the SNP regression coefficient *β*_i_ and the Bayes factor estimates.** Left panels: Manhattan plots of the genome-environment association results for the 6 climate variables. The x-axis indicates the position along each chromosome. The five chromosomes are presented in a row along the x-axis in different degrees of blue. The y-axis indicates either the posterior mean of the regression coefficient *β*_i_ (M_Beta value) or the Bayes factor (BF_mc_ expressed in deciban units), estimated by the AUX model implemented in the program BayPass. Right panels: Estimates of the Bayes factor as a function of the SNP regression coefficients (*β*_i_) for the 6 climate variables.


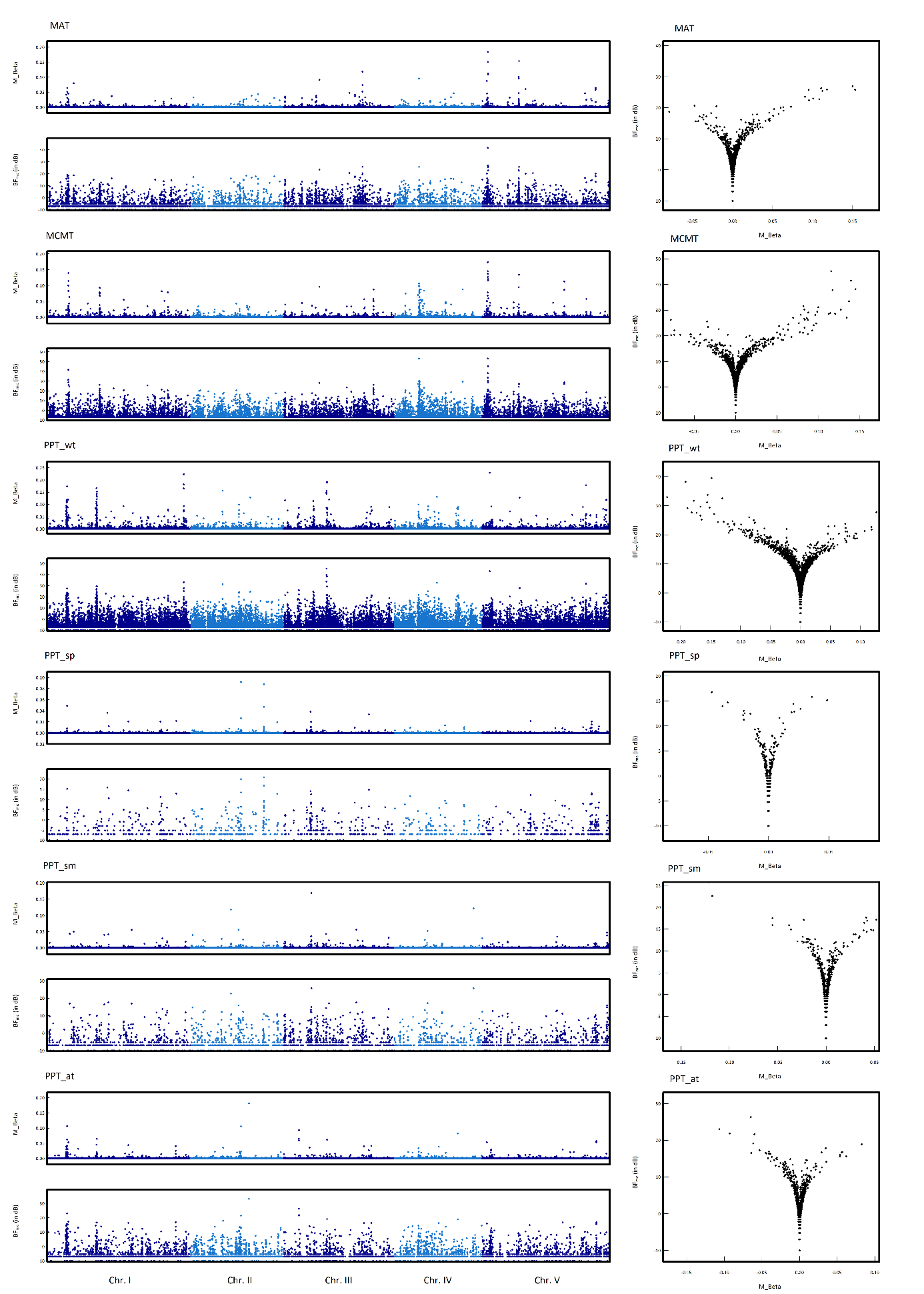


**Figure S5. Alignment of RZ-1c proteins in the family Brassicaceae.** Numbering corresponds to the amino acid sequence. The asterisk indicates the position of the replacement observed in the conserved DRY-motif in the Midi-Pyrénées region (position 2013 in the amino acid sequence of *A. thaliana*). RZ_1C_Col-0: reference accession Col-0 from *A. thaliana*, RZ_1C_MP: alternative protein sequence observed in the Midi-Pyrénées region, A.lyrata: *Arabidopsis lyrata*, A.halleri: *Arabidopsis halleri*, B.stricta: *Boechera stricta*, C.rubella: *Capsella rubella*, C.grandiflora: *Capsella grandiflora*, B.oleracea: *Brassica oleracea*, B.rapa: *Brassica rapa*.

**
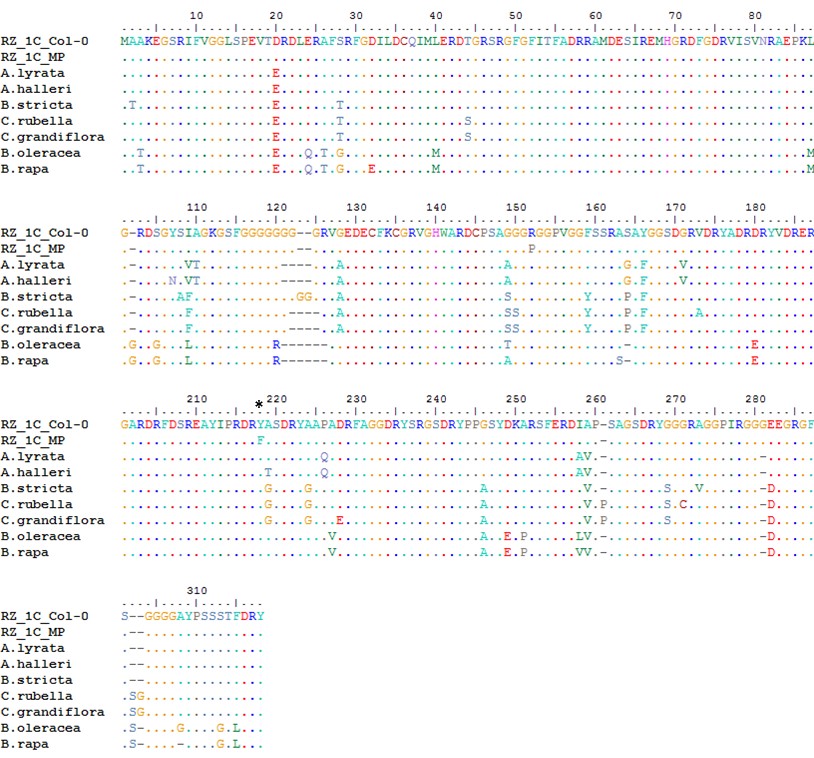
**
